# Endothelial Akt1 loss promotes prostate cancer metastasis via β-catenin-regulated tight-junction protein turnover

**DOI:** 10.1038/s41416-018-0110-1

**Published:** 2018-05-14

**Authors:** Fei Gao, Abdulrahman Alwhaibi, Sandeep Artham, Arti Verma, Payaningal R. Somanath

**Affiliations:** 1 Clinical and Experimental Therapeutics, University of Georgia, Augusta, GA 30912 USA; 2 Charlie Norwood VA Medical Center, Augusta, GA 30912 USA; 3 Department of Urology, The First Affiliated Hospital, Chongqing University, Chongqing, China; 4 Department of Medicine, Vascular Biology Center and Georgia Cancer Center, Augusta University, Augusta, GA 30912 USA

**Keywords:** Experimental models of disease, Metastasis

## Abstract

**Background:**

Cancer research, in general, is focused on targeting tumour cells to limit tumour growth. These studies, however, do not account for the specific effects of chemotherapy on tumour endothelium, in turn, affecting metastasis.

**Methods:**

We determined how endothelial deletion of Akt1 promotes prostate cancer cell invasion in vitro and metastasis to the lungs in vivo in endothelial-specific Akt1 knockdown mice.

**Results:**

Here we show that metastatic human PC3 and DU145 prostate cancer cells invade through Akt1-deficient human lung endothelial cell (HLEC) monolayer with higher efficiency compared to control HLEC. Although the endothelial Akt1 loss in mice had no significant effect on RM1 tumour xenograft growth in vivo, it promoted metastasis to the lungs compared to the wild-type mice. Mechanistically, Akt1-deficient endothelial cells exhibited increased phosphorylation and nuclear translocation of phosphorylated β-catenin, and reduced expression of tight-junction proteins claudin-5, ZO-1 and ZO-2. Pharmacological inhibition of β-catenin nuclear translocation using compounds ICG001 and IWR-1 restored HLEC tight-junction integrity and inhibited prostate cancer cell transendothelial migration in vitro and lung metastasis in vivo.

**Conclusions:**

Here we show for the first time that endothelial-specific loss of Akt1 promotes cancer metastasis in vivo involving β-catenin pathway.

## Introduction

Currently, research in the development of cancer therapy more focused on the pathways promoting tumour cell growth and invasion. Studies that address the specific role of a pathway in stromal cells and how drugs affect stroma when used for cancer therapy are fewer. Among the cells in the tumour microenvironment, tumour endothelium plays a significant role not only in tumour angiogenesis, perfusion and metastasis^1–3^ but also as the first line of defense in a patient’s fight against cancer cell metastasis to other vital organs. Hence, it is important to determine the specific role of a pathway and the effect of a drug on tumour vasculature alone so as to improve the efficacy and minimise the side effects of cancer chemotherapy.

Preclinical and clinical research evidence has revealed the integral role of phosphatase and tensin homologue (PTEN)-Akt pathway in multiple cancers,^4^ including prostate cancer.^5^ A number of studies from our laboratory have indicated that pharmacological and genetic inhibition of Akt, particularly Akt1, inhibits prostate and bladder cancer cell function in vitro and tumour xenograft growth in vivo.^6–8^ We previously reported that, drugs such as statins and angiotensin receptor blocker candesartan, that have the ability to normalise Akt1 activity in prostate cancer by inhibiting hyperactive Akt1 in prostate cancer cells,^9–11^ and activating Akt1 from its basal state in endothelial cells, led to the inhibition of prostate cancer cell transendothelial migration in vitro.^12^ We have also reported that Akt1 gene knockout in mice promoted tumour vascular permeability and angiogenesis in a murine B16F10 melanoma model.^13^ Most recently, we demonstrated that endothelial-specific knockdown of Akt1 results in increased vascular permeability via FoxO- and β-catenin-mediated suppression of endothelial tight-junction claudin expression, mainly claudin-5.^14^ Since many inhibitors of Akt are in different phases of clinical trials for various types of cancers, it is important to understand the effect of Akt1 suppression in endothelial cells of tumour vasculature, and its consequences on tumour growth and metastasis.

In the current study, we investigated the effects of endothelial-specific knockdown of Akt1, a major endothelial isoform of Akt^13^ on prostate cancer cell invasion in vitro and metastasis in vivo using murine lung colonisation model of in vivo metastasis. Our analysis revealed that Akt1 deficiency in human lung microvascular endothelial cells (HLECs) enhances the ability of human metastatic PC3 and DU145 prostate cancer cells to migrate across the endothelial monolayer in vitro, and murine RM1 prostate cancer cell metastasis to the lungs in vivo, with no changes in the growth of RM1 tumour xenografts in vivo. The akt1 loss in HLECs resulted in increased translocation of phosphorylated β-catenin from the endothelial-barrier junctions to the cytosol and the nucleus, in turn, suppressing the transcription of endothelial tight-junction proteins such as claudin-5, ZO-1 and ZO-2. Pharmacological inhibition of β-catenin in HLEC with ICG001 and IWR-1 restored the tight-junction protein expression and inhibited DU145 cell transendothelial migration in vitro and murine RM1 cell lung metastasis in vivo. Although Akt1 is a well-known mediator of oncogenic transformation^15^ and prostate tumour growth,^6, 8^ our current study demonstrates for the first time that endothelial-specific Akt1 loss will promote prostate cancer metastasis via nuclear translocation of β-catenin and suppression of endothelial tight-junction protein expression.

## Materials and methods

### Generation of ‘VECad-Cre-Akt1’ transgenic mouse model

All the mouse experiments were performed with the approval of Charlie Norwood Veterans Affairs Medical Center Institutional Animal Care and Use Committee (approval reference #13-09-062). All studies involving animals are reported in accordance with the ARRIVE guidelines for reporting experiments involving animals. Carbon dioxide asphyxiation followed by cervical dislocation was performed for killing. Isoflurane inhalation was used for anaesthesia. For our study, we utilised an endothelial-specific, tamoxifen-inducible Akt1 knockout mouse model (VECad-Cre-Akt1) by crossing Akt1 *LoxP* mice with VE-Cadherin-*Cre* mice in the pure C57BL6 background as reported previously.^14^ Age-matched 8- to 12-week-old male mice were used in the study. Genotyping was performed using specific primers for A1-3Loxp: TCACAGAGATCCACCTGTGC, and A1-4113R: GCAGCGGATGATAAAGGTGT. Tamoxifen (Sigma, St. Louis, MO) stock solution (100 mg/ml) was prepared to dissolve in absolute ethanol and stored in an aluminum foil-covered plastic tubes at −20 °C. Right before injection, sterile corn oil was added to dilute tamoxifen to a final concentration of 10 mg/ml. Tamoxifen (1 mg/10 g dose) was administered to the mice using a 27 G needle via intraperitoneal (i.p.) injection every 24 h for 5 consecutive days. Following this, mice were maintained on a custom-made Tamoxifen diet (Harlan, Madison, WI) for the duration of the experiments.

### Cell culture and preparation of shAkt1 stable cell lines

Primary HLECs (CC-2527; Lonza, Allendale, NJ) were maintained in EBM-2 medium with a GM-2 Bullet Kit (Lonza; Walkersville, MD). All cultures were maintained in a humidified 5% CO_2_ incubator at 37 °C and routinely passaged when 80–90% confluent. Stable shControl and shAkt1 (ACGCTTAACCTTTCCGCTG) HLEC cells were generated using SMART vector 2.0 lentivirus particles (10^9^ p.f.u.) (Thermo Scientific, Waltham, MA). Lentiviral particles were mixed in 1 ml Hyclone SFM4Transfx-293 (Fisher, Hanover Park, IL) and added along with 1 µl Polybrene (10 mg/ml, American Bioanalytical, Natick, MA). Three days later, transfection efficiency was tested through Turbo-GFP expression and subjected to 4 µg/ml puromycin (Life Technologies, Grand Island, NY) selection until all the cells expressed GFP. Pharmacological inhibition of Akt activity (phosphorylation) was achieved by treating the cells for 24 h with 10 µM MK-2206, an Akt inhibitor widely used in the clinical trials and GSK-3 inhibition was achieved by using 10 µM SB415286 (Selleckchem, Houston, TX).

### Immunofluorescence staining and confocal imaging

Immunofluorescent staining of shControl and shAkt1 HLEC monolayers was performed using the chamber slides (Fisher, Hanover Park, IL). HLEC were cultured to the monolayer, treated with either DMSO, 10 µM ICG001 or 10 µM IWR-1 for 24 h and washed twice with ice-cold PBS, fixed using 2% paraformaldehyde for 30 min, permeabilised with 0.1% Triton X-100 for 15 min and blocked with 2% BSA in sterile PBS for 1 h. Cell monolayers were then incubated with antibodies against claudin-5, pS33/S37/T41-β-catenin, total β-catenin, ZO-1 and ZO-2 (1:100, Rabbit antibodies, Cell Signaling, Danvers, MA) at 4 °C overnight. Immunofluorescence was revealed using goat anti-rabbit AlexaFlour-488 secondary antibodies (1:2000, Life Technologies). Cells were mounted on a glass slide using DAPI containing mounting medium (Vector Laboratories, PA). Images were captured using a confocal microscope (LSM510, Zeiss, Germany). The ‘blue’ nuclear staining of DAPI was pseudo-coloured to ‘green’ for a better contrast to detect co-localisation with β-catenin. Controls with no primary antibodies were analysed for any non-specific staining. For immunofluorescence staining of lung sections, lungs were fixed in 4% paraformaldehyde overnight and frozen sections were probed for Akt1 and CD31, followed by AlexaFlour secondary antibodies and mounted on DAPI. Images were taken with a fluorescent microscope (Axiovert100M, Zeiss). All controls gave negative results with no detectable non-specific labelling.

### Western blot analysis

Cell lysates were prepared using complete lysis buffer (EMD Millipore, San Diego, CA) with protease and phosphatase inhibitor cocktails (Roche Diagnostics, Indianapolis, IN). Protein quantification was performed using Dc protein assay (Bio-Rad, Hercules, CA). Western blot analysis was performed as described previously.^16, 17^ Antibodies used include Akt1, Akt2, Akt3, pan-Akt, ^Tyr216^GSK-3β, ^Ser33/37/Thr41^β-catenin, claudin-5, ZO-1 and ZO-2 (Cell Signaling) and anti-β-actin (Sigma). Densitometry was done using NIH Image J software.

### RNA isolation, cDNA preparation and qRT-PCR

Cells were grown until confluent in six-well culture plates and treated with DMSO and β-catenin inhibitors 10 µM ICG001 or 10 µM IWR-1 for 24 h. RNA was extracted using RNA isolation kit (RNeasy Plus, Qiagen, Valencia, CA) and RNA quality was confirmed using Nanodrop 2000 spectrophotometer (Thermo Scientific). Complementary DNA (cDNA) was synthesised from 500 ng of RNA using RT2 First Strand kit (Qiagen) using a StepOne Plus thermal cycler and detection software (Applied Biosystems, Foster City, CA) and quantitative real-time PCR (qRT-PCR) was performed using the RT^2^ SYBR Green ROX qPCR Mastermix (Qiagen) in a real-time PCR equipment (Applied Biosystems). Sample cDNA was amplified and quantified over a large number of shorter cycles under the following conditions: an initial 10 min 95 °C period followed by 40 cycles of 95 °C for 15 s, 60 °C for 1 min and 72 °C for 15 s. To analyse the fluorescence signal, a threshold cycle (Ct) was determined, using the exponential growth phase and the baseline signal from fluorescence vs cycle number plots. The following primers were used for the messenger RNA (mRNA) analysis: claudin-5 (forward: 5′-CTGCTGGTTCGCCAACATT-3′ and reverse: 5′-TGCGACACGGGCACAG-3′), ZO-1 (forward: 5′-CAGCCGGTCACGATCTCCT-3′ and reverse: 5′-TCCGGAGACTGCCATTGC-3′), ZO-2 (forward: 5′-TTGAAGACACGGACGGTGAA-3′ and reverse: 5′-GTGATGGACGACACCAGCG-3′), Axin-2 (forward: 5′-GACGGACAGCAGTGTAGATG-3′ and reverse: 5′-GGGTTCTCGGGAAATGA-3′) and GAPDH (forward: 5′-AGCCACATCGCTCAGACAC-3′ and reverse: 5′-GCCCAATACGACCAAATCC-3′). Ct values were normalised to the level of expression of the housekeeping gene (GAPDH).

### Transwell invasion assay

24-well Transwell permeable plates with 8.0 µm polycarbonate membrane coated with Matrigel (Corning, Tewksbury, MA) were used. Briefly, cells were seeded in six-well plates and treated with either DMSO or ICG001 (10 µM) every day for a total of 72 h. Following this, cells were detached using cell dissociation buffer (sterile 20 mM EDTA in PBS [pH 7.4]) and 5000 cells were plated on the upper chamber of the transwell plates. Upper chambers were filled with serum-free medium and lower chambers were filled with DMEM/HG medium containing 20% FBS as a chemoattractant. Cells that invaded the matrigel and reached the bottom layers of the top chambers after 24 h of incubation were fixed using 3.7% paraformaldehyde then stained with 0.5% crystal violet solution. The cells were counted manually using the inverted microscope and the average was calculated from four random images.

### In vivo mouse tumour xenograft model

Murine RM1 metastatic prostate cancer cells were grown to confluence in 75-ml flasks and were re-suspended in PBS, counted and an equal number of cells (1 × 10^6^) was injected subcutaneously in 7- to 8-week-old C57BL6 mice. Mice were killed on day 16, and tumours were dissected, weighed, and tissues were fixed in 4% PFA for immunohistochemistry analysis.

### In vivo mouse model of prostate cancer colonisation to lungs

Eight-week-old wild-type and VECad-Cre-Akt1 mice were divided into two groups for the administration of RM1 cells. Cells (0.5 × 10^6^) suspended in normal saline were injected into each group of mice separately via the tail vein. Mice were treated with DMSO (control) or ICG001 (50 mg/kg body weight). In all the experiments, mice were evaluated for the presence of metastases 16 days after cell administration. 1.5 ml of 15% India-ink solution was injected intratracheally to stain the lungs and visualise the non-stained tumour nodules. Stained lungs were carefully resected and rinsed in Fekete’s solution (300 ml 70% ethanol, 30 ml 37% formaldehyde, 5 ml glacial acetic acid), then placed in fresh Fekete’s solution overnight. The whole lungs were collected for the counting of tumour nodules. Following this, mice lungs were subjected to equal interval cross section (2 mm) analysis for RM1 cell colonisation to the lung tissues. Tumour area was measured using the NIH Image J software.

### Statistical analysis

All the data are presented as mean ± SD and were calculated from multiple independent experiments performed in quadruplicates. For normalised data analysis, data was confirmed that normality assumption was satisfied and analysed using paired sample *t-*test (dependent *t-*test) and/or further confirmed with non-parametric test and Wilcoxon signed-rank test. For all other analysis, Student’s two-tailed *t*-test or analysis of variance test were used to determine significant differences between treatment and control values using the GraphPad Prism 4.03 software and SPSS 17.0 software.

## Results

### Endothelial Akt1 loss promotes transendothelial migration and invasion of human prostate cancer cells

**Fig. 1:**
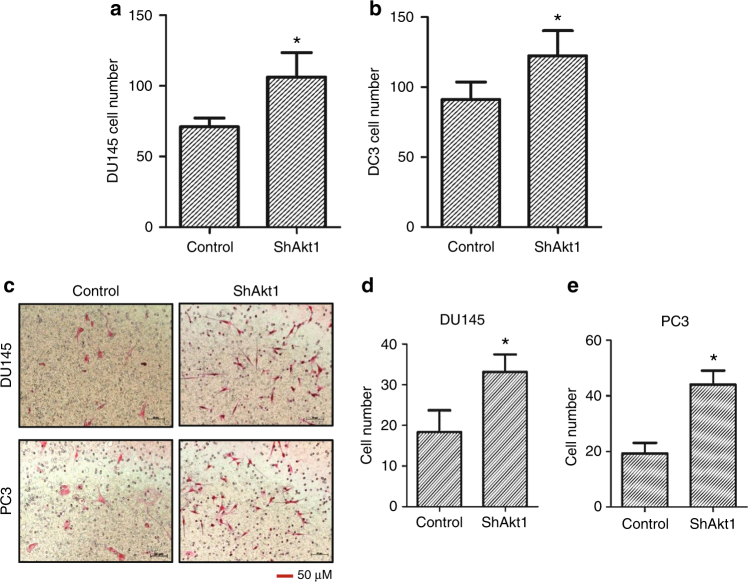
PC3 and DU145 cells interact and invade through Akt1-deficient HLEC monolayer compared to Akt1 intact control. **a**, **b** Bar graphs showing increased attachment of PC3 and DU145 cells to Akt1-deficient HLEC monolayer compared to control (*n* = 6). **c** Representative images of transwell plates showing DU145 and PC3 cells successfully invaded through the Matrigel and control as well as Akt1-deficient HLEC monolayers. **d**, **e** Bar graphs showing increased invasion of DU145 and PC3 cells through Akt1-deficient HLEC monolayers compared to control (*n* = 6). Data are represented as mean ± SD. *p < 0.01, scale bar: 50 µm

Whereas the role of Akt in oncogenic transformation and tumour progression in various cancers is well known, the specific effect of Akt1 activity modulation in endothelial cells on tumour growth and metastasis is not known. Hence, utilising shControl and shAkt1 primary HLEC, we investigated the effects of endothelial Akt1 knockdown on PC3 and DU145 cell transendothelial migration in vitro. Our study indicated that loss of Akt1 in HLEC resulted in the enhanced interaction of PC3 and DU145 cells compared to Akt intact HLEC as evidenced by their Turbo-GFP expression (Fig. 1a, b). Similarly, both PC3 and DU145 cells were able to transmigrate through ShAkt1 HLEC monolayer than a shControl HLEC monolayer (Fig. 1c–e), thus indicating that endothelial Akt1 loss augments prostate cancer cell transendothelial migration.

### Endothelial Akt1 loss results in β-catenin translocation from the barrier junctions to the cytosol and nucleus

**Fig. 2:**
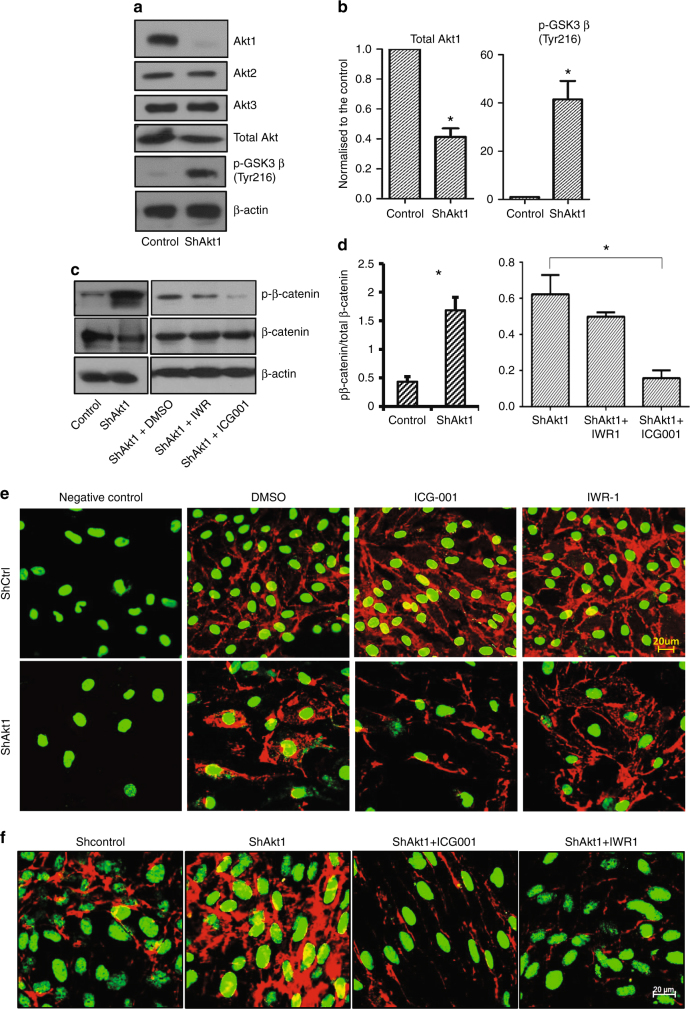
Akt1 gene silencing in HLEC increases β-catenin phosphorylation and nuclear localisation associated with a decrease in β-catenin at the HLEC-barrier. **a** Representative western blot images showing expression levels of Akt isoforms, total Akt, ^Tyr216^GSK-3β, pβ-catenin and total β-catenin in shControl and shAkt1 HLEC lysates. **b** Bar graphs of western blot band densitometry showing changes in the expression levels of Akt1, ^Tyr216^GSK-3β and pβ-catenin in shControl and shAkt1 HLEC lysates (*n* = 4). **c**, **d** Representative western blot images and bar graph of western blot band densitometry showing changes in the expression levels of pβ-catenin normalised to total β-catenin in ShAkt1 HLEC lysates upon treatment with β-catenin inhibitors ICG001 and IWR-1 (*n* = 4). **e** Representative confocal images of β-catenin and DAPI in ShAkt1 HLEC monolayers treated with control DMSO and β-catenin inhibitors (ICG001 and IWR-1) compared to the negative control where non-immune serum was used as a control (*n* = 6). Red indicates total β-catenin and green indicates DAPI staining. **f** Representative confocal images of pβ-catenin and DAPI-stained ShAkt1 HLEC monolayers treated with control DMSO and β-catenin inhibitors (ICG001 and IWR-1) (*n* = 6). Red indicates total pβ-catenin and green indicates DAPI staining. Data are represented as mean ± SD. *p < 0.05

To investigate the downstream mediator of enhanced transendothelial migration and lung colonisation by prostate cancer cells with the endothelial Akt1 loss, we determined the role of endothelial GSKβ-β-catenin pathway in this process. In our analysis, Akt1 gene silencing did not affect the expression levels of Akt2 and Akt3 isoforms (Fig. 2a). Interestingly, Akt1 gene knockdown resulted in increased activating phosphorylation of GSK3β at Tyr216 residue (Fig. 2a, b), likely due to activation of Src.^18^ Endothelial Akt1 gene silencing was also associated with decreased phosphorylation of Akt and increased phosphorylation of β-catenin, a GSK3β substrate (Fig. 2a, b), which was reversed upon pharmacological inhibition of β-catenin using ICG001 or IWR-1 (Fig. 2c, d). Interestingly, Akt1 gene silencing in HLEC resulted in the increased presence of β-catenin in the cytosol and nucleus from its normal localisation in the endothelial adherens junctions, and treatment with ICG001 and IWR-1 that prevent β-catenin nuclear localisation restored β-catenin expression in the cell–cell junctions (Fig. 2e; Supplemental Fig. 1A). Immunostaining with pS33/S37/T41-β-catenin antibodies further confirmed the presence of phosphorylated β-catenin in the cytosol and nucleus of ShAkt1 HLECs compared to ShControl cells, which was reversed with pharmacological inhibition of β-catenin (Fig. 2f; Supplemental Fig. 1B). Together, these data indicate that inhibition of β-catenin nuclear translocation in endothelial cells may limit prostate cancer transendothelial migration and hence metastasis.

### Reduced tight-junction protein and mRNA expression in Akt1-deficient HLEC is rescued with pharmacological inhibition of β-catenin

**Fig. 3:**
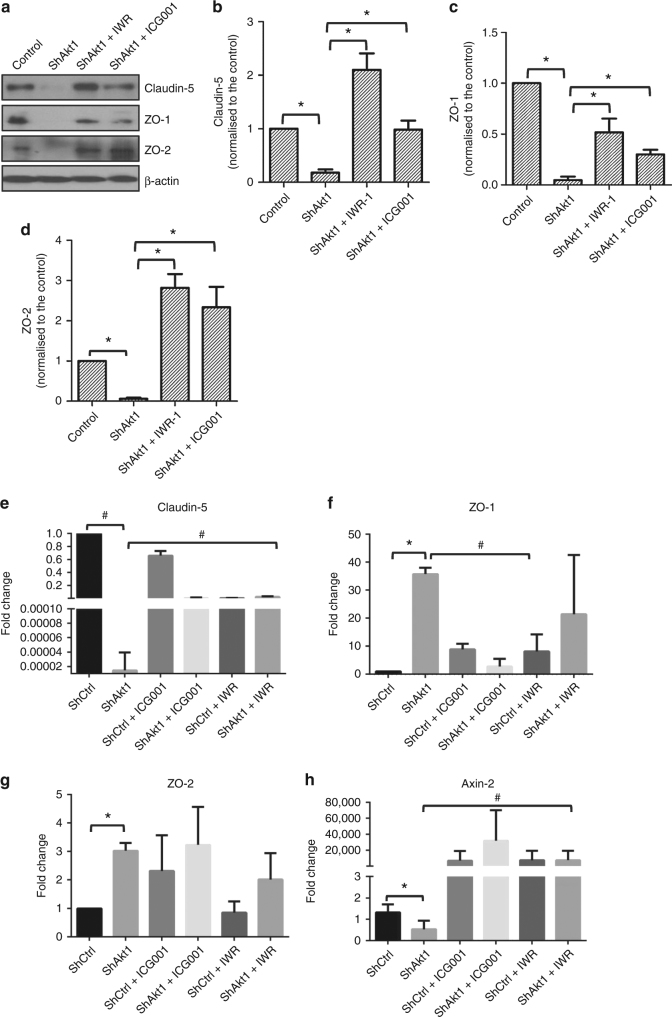
Pharmacological inhibition of β-catenin restores tight-junction protein expression in Akt1 knockout HLEC. **a** Representative western blot images showing expression levels of tight-junction proteins claudin-5, ZO-1 and ZO-2 compared to β-actin in shControl and shAkt1 HLEC lysates. **b**–**d** Bar graphs of western blot band densitometry showing changes in the expression levels of tight-junction proteins claudin-5, ZO-1 and ZO-2, respectively, and normalised to β-actin in shControl and shAkt1 HLEC lysates (*n* = 3). **e**–**h** Bar graphs of qRT-PCR analysed mRNA expression changes in tight-junction proteins claudin-5, ZO-1 and ZO-2, respectively, along with Axin-2, a positive control to confirm β-catenin transcriptional activity in shControl and shAkt1 HLECs (*n* = 6). Data are represented as mean ± SD. *p < 0.05; ^#^*p* < 0.01

We determined whether β-catenin is involved in the transcriptional regulation of endothelial tight-junction proteins. To do this, we performed western blot analysis for protein analysis, qRT-PCR for mRNA analysis and immunocytochemistry for localisation analysis in ShControl and ShAkt1 HLECs in vitro in the presence and absence of β-catenin inhibitors. In our western blot analysis, Akt1 gene silencing in HLEC resulted in decreased expression of claudin-5, ZO-1 and ZO-2 (Fig. 3a–d), which are integral in the formation of tight-junction complex. In agreement with the protein expression analysis, qRT-PCR analysis also indicated a significant decrease in the claudin-5 mRNA levels in shAkt1 HLEC compared to shControl, which was reversed upon treatment with β-catenin inhibitors ICG-001 and IWR-1 (Fig. 3e). Our subsequent analysis of Axin-2 mRNA expression levels in shAkt1 cells compared to shControl as well as its modulation with ICG-001 and IWR-1 treatment further confirmed the involvement of β-catenin in the process (Fig. 3h). Surprisingly, ZO-1 and ZO-2 mRNA levels with regard to Akt1 expression and treatment with β-catenin inhibitors were not similar to claudin-5 (Fig. 3f, g). Interestingly, an increase or no significant change in their mRNA levels (Fig. 3f, g) was observed in ShAkt1 cells compared to ShControl.

**Fig. 4:**
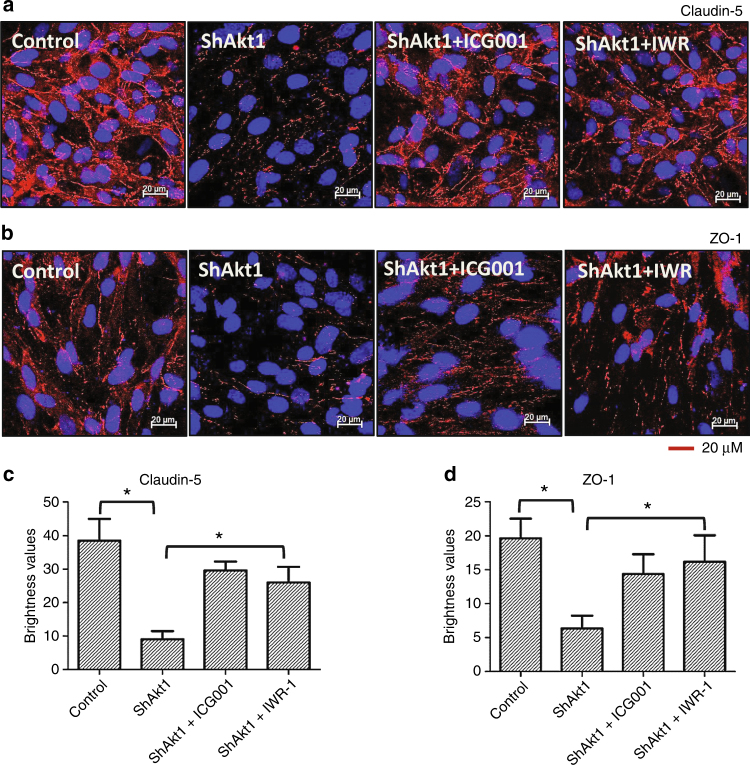
Pharmacological inhibition of β-catenin restores tight-junction protein expression in Akt1 knockout HLEC. **a**, **b** Representative confocal images of shControl HLEC, shAkt1 HLEC, shAkt1 HLEC treated with ICG001 and shAkt1 HLEC treated with IWR-1 showing changes in the expression levels of tight-junction proteins claudin-5 and ZO-1, respectively. **c**, **d** Bar graphs of quantitative analysis of changes in the expression levels of tight-junction proteins claudin-5 and ZO-1, respectively, in shControl HLEC, shAkt1 HLEC, shAkt1 HLEC treated with ICG001 and shAkt1 HLEC treated with IWR-1 (*n* = 4). Data are represented as mean ± SD. *p < 0.05

These results in the mRNA and protein expression of endothelial tight-junction molecules with respect to Akt1 and β-catenin activities were also confirmed by immunocytochemistry (Fig. 4a–d). Interestingly, treatment with β-catenin inhibitors restored expression of these tight-junction proteins in Akt1-deficient HLEC (Fig. 3a–d) in the barrier junctions (Fig. 4a–d). This indicates that nuclear β-catenin in Akt1-deficient HLEC functions as a transcriptional repressor for the endothelial tight-junction proteins such as claudin-5, ZO-1 and ZO-2.

### Pharmacological inhibition of Akt inhibits endothelial tight-junction protein expression

**Fig. 5:**
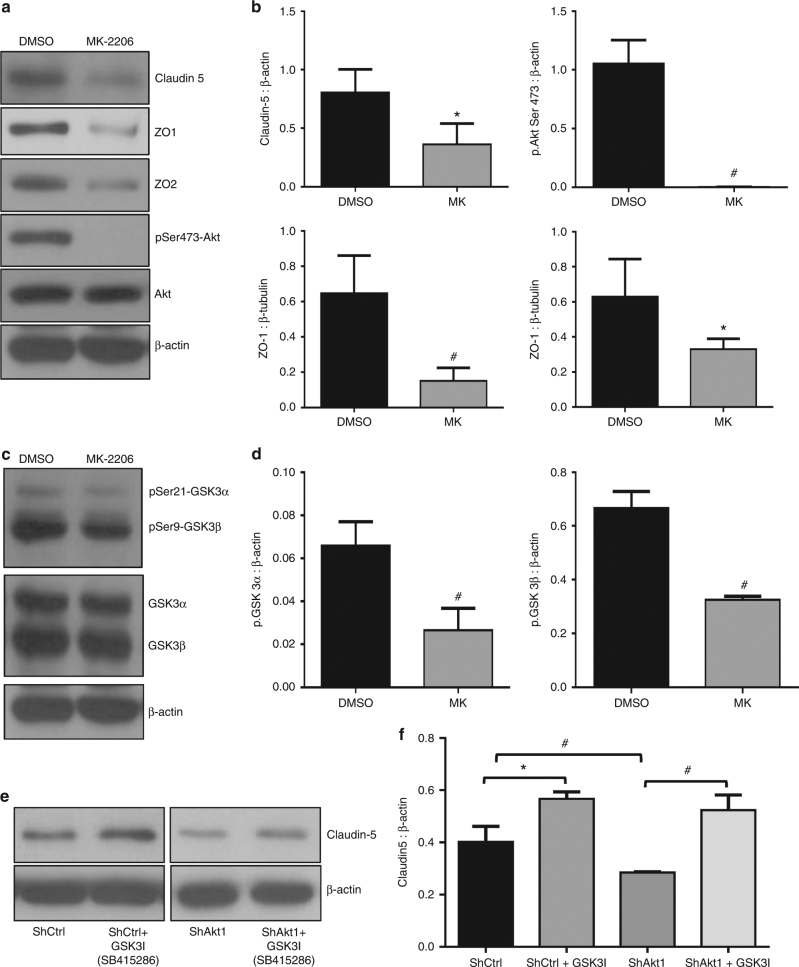
Pharmacological inhibition of Akt reduces the expression of tight-junction proteins claudin-5, ZO-1, ZO-2 in HLEC. **a** Representative western blot images showing expression levels of tight-junction proteins claudin-5, ZO-1 and ZO-2 compared to β-actin in shControl and shAkt1 HLEC lysates. **b** Bar graphs of western blot band densitometry showing changes in the expression levels of tight-junction proteins claudin-5, ZO-1 and ZO-2, and phosphorylated Akt, normalised to β-actin in shControl and shAkt1 HLEC lysates (*n* = 3). **c** Representative western blot images showing expression levels of phosphorylated GSK3α/β compared to total GSK3α/β and β-actin in shControl and shAkt1 HLEC lysates. **d** Bar graphs of western blot band densitometry showing changes in the decreased expression levels of phosphorylated GSK3α/β normalised to β-actin in shControl and shAkt1 HLEC lysates (*n* = 3). **e** Representative western blots showing increased expression levels of claudin-5 compared to β-actin in shControl and shAkt1 HLEC lysates in the presence and absence of GSK3 inhibitor SB415286. **f** Bar graphs of western blot band densitometry showing changes in the expression levels claudin-5 compared to β-actin in shControl and shAkt1 HLEC lysates in the presence and absence of GSK3 inhibitor SB415286. (*n* = 3). Data are represented as mean ± SD. *p < 0.05; ^#^*p* < 0.01

Treatment with 10 µM MK-2206, an Akt inhibitor widely used in the clinical trials for cancer indicated a significant decrease in the expression of phosphorylated Akt, Phosphorylated GSK-3α/β, claudin-5, ZO-1 and ZO-2 protein levels compared to DMSO-treated controls in HLECs (Fig. 5a–d). In order to confirm the role of GSK-3 in the process, we also performed the effect of panGSK-3 inhibitor 10 µM SB415286 on claudin-5 expression. Our analysis indicated that suppression of GSK-3 activity resulted in significantly increased expression of claudin-5 in HLECs (Fig. 5e, f), thus demonstrating the involvement of Akt1-GSK-3 pathway in the regulation of claudin-5 expression in endothelial cells.

### Pharmacological inhibition of β-catenin limits DU145 prostate cancer cell invasion in vitro

**Fig. 6:**
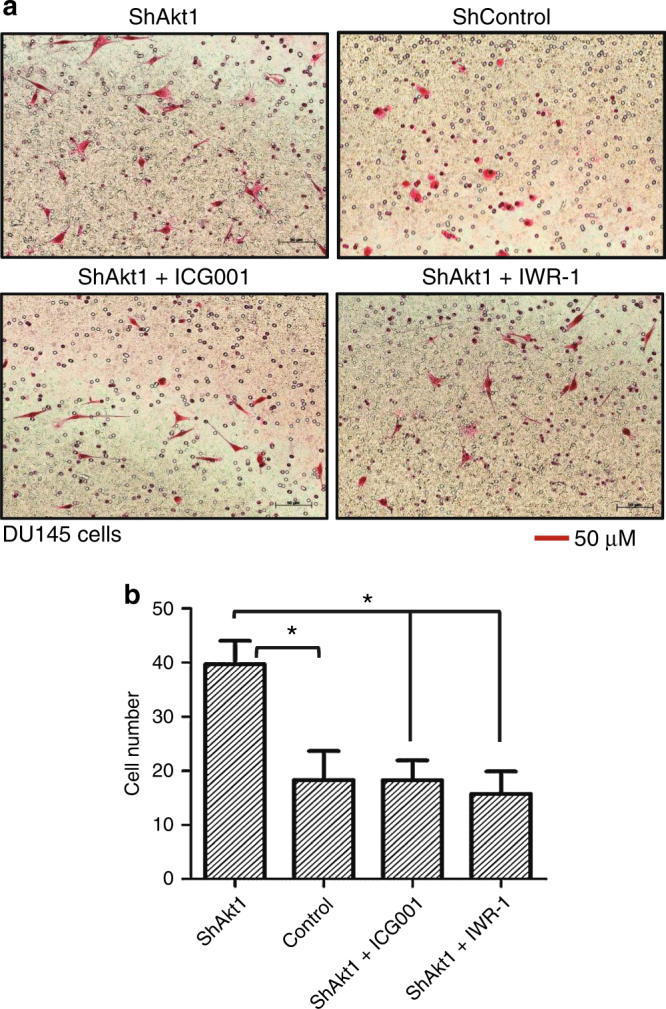
Endothelial Akt1 loss promotes invasion of human DU145 prostate cancer cells in vitro. **a** Representative Images of transwell plates showing DU145 cells successfully invaded through the Matrigel and Akt1-deficient HLEC monolayers alone and after treatment with ICG001 and IWR-1. **b** Bar graphs showing invaded DU145 cells through Akt1-deficient HLEC monolayers in the presence and absence of treatment with β-catenin inhibitors ICG001 and IWR-1 (*n* = 6). Data are represented as mean ± SD. *p < 0.05

Next, we determined whether pharmacological inhibition of β-catenin would inhibit enhanced transendothelial migration of human DU145 prostate cancer cells through Akt1-deficient HLEC monolayer in vitro and limit lung colonisation of murine RM1 prostate cancer cells in VECad-Cre-Akt1 mice in vivo. In our study, treatment of ShAkt1 HLEC monolayers with β-catenin inhibitors ICG001 and IWR-1 resulted in significant inhibition of transendothelial migration of human DU145 prostate cancer cells as compared to DMSO-treated ShAkt1 HLEC controls (Fig. 6a, b). Thus, our data indicate that nuclear β-catenin level in endothelial cells is an important determinant of prostate cancer cell invasion in vitro.

### Endothelial Akt1 deletion in mice does not modulate the growth of RM1 tumour xenografts but promotes RM1 cell metastasis to the lungs via β-catenin

**Fig. 7:**
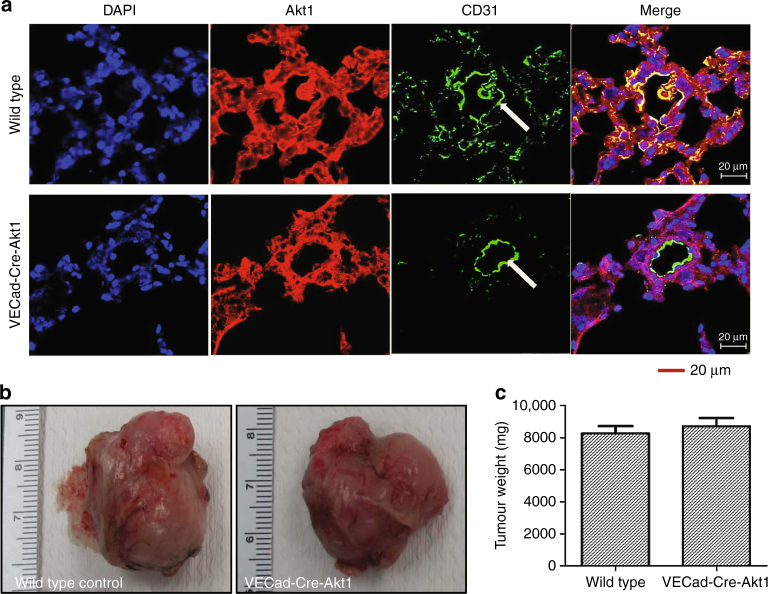
Endothelial Akt1 loss has no effect on the growth of prostate tumour xenografts in vivo. **a** Representative confocal images of lung sections from wild-type and VECad-Cre-Akt1 mice stained with Akt1, CD31, and DAPI showing loss of Akt1 in VECad-Cre-Akt1 lungs. **b** Representative images of RM1 murine prostate cancer tumour xenografts collected from wild-type and VECad-Cre-Akt1 mice. **c** Bar graph showing quantification of the size of RM1 tumour xenografts collected from wild-type and VECad-Cre-Akt1 mice (*n* = 6). Data are represented as mean ± SD. Scale bar: 20 µm

**Fig. 8:**
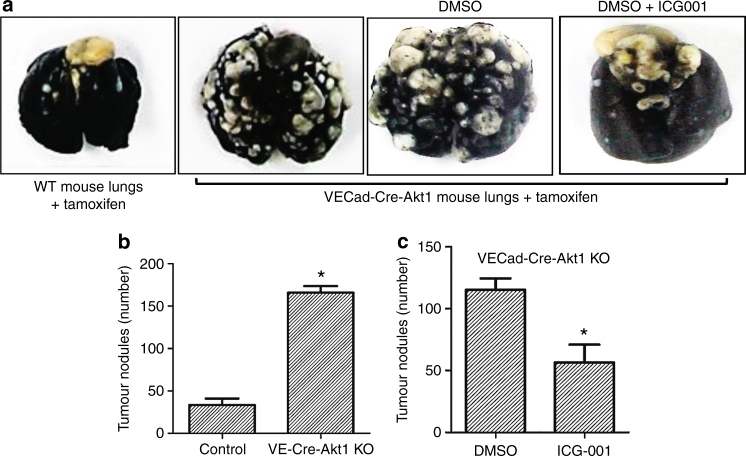
Endothelial Akt1 loss promotes metastasis of murine RM1 prostate cancer cells in vivo and is reversed by the pharmacological inhibition of β-catenin. **a** Representative images of lungs collected from wild-type, VECad-Cre-Akt1, VECad-Cre-Akt1 mice treated with DMSO and VECad-Cre-Akt1 mice treated with β-catenin inhibitor ICG001 showing RM1 murine prostate cancer metastatic nodules. **b** Bar graph showing quantification of the number of RM1 tumour nodules comparing the lungs of wild-type mice with VECad-Cre-Akt1 mice (*n* = 5). **c** Bar graph showing quantification of the number of RM1 tumour nodules comparing the lungs of VECad-Cre-Akt1 mice treated with DMSO with VECad-Cre-Akt1 mice treated with β-catenin inhibitor ICG001 (*n* = 5). Data are represented as mean ± SD. *p < 0.05

In order to study the effects of endothelial Akt1 suppression on prostate cancer cell metastasis in vivo, we utilised a tamoxifen-inducible, endothelial-specific Akt1-deficient mouse model (Fig. 7a) and murine RM1 metastatic prostate cancer cell line of C57/BL6 origin. This combination also has the advantage of not compromising the immune system when immune-compromised mice are used for lung colonisation studies. Subcutaneous implantation of murine RM1 prostate tumours showed no significant difference in weight between tamoxifen-treated wild-type and VECad-Cre-Akt1 mice (Fig. 7b, c). Interestingly, our analysis of lung colonisation of murine RM1 cell lines intravenously administered to the mice through tail vein injection^19^ revealed that endothelial Akt1 loss in VECad-Cre-Akt1 mice promoted RM1 cell metastasis to the lungs by fivefold as compared to tamoxifen wild-type mice controls (Fig. 8a, b). Next, we determined whether pharmacological inhibition of β-catenin would inhibit enhanced transendothelial migration of human DU145 prostate cancer cells through Akt1-deficient HLEC monolayer in vitro and limit lung colonisation of murine RM1 prostate cancer cells in VECad-Cre-Akt1 mice in vivo. In our study, VECad-Cre-Akt1 mice treated with ICG001 had a significantly reduced lung colonisation of RM1 cells compared to the DMSO-treated control animals (Fig. 8a, c). Thus, our data show that endothelial Akt1 knockdown, although does not modulate prostate tumour growth, it promotes prostate cancer metastasis to the lungs, and that nuclear β-catenin level in endothelial cells is an important determinant of prostate cancer lung colonisation in vivo.

## Discussion

Vascular permeability is the hallmark of several diseases including cancer and organ injuries.^20^ Although numerous signalling pathways are involved in the regulation of endothelial-barrier function, PI3K-Akt and Src pathways are of prime importance in regulating endothelial activation, barrier function and gene expression.^13, 14, 21–23^ A variety of stimuli including growth factors, cytokines, vascular permeability-inducing agents like vascular endothelial growth factor (VEGF), and barrier protective agents like angiopoietin-1 induces activation of Akt and Src and hence they are greatly implicated in the regulation of vascular wall integrity.^24, 25^ While there have been contradicting reports about the role of Akt in endothelial-barrier regulation, our recent studies have shown the integral role of Akt1^14^ and its cross-talk with Src^21^ in the long-term protection of endothelial barrier in response to VEGF and angiopoietin-1. Although a number of clinical trials have been performed on the use of Akt inhibitors for cancer therapy, none has been approved so far and many studies have shown to further worsen the condition.^26–31^ As of today, the role of endothelial Akt1 in cancer metastasis and the effect of endothelial Akt1 loss on tumour growth and metastasis have not been studied.

A major finding from the current study is that the lack of Akt1 in HLECs promotes prostate cancer cell transendothelial migration in vitro and metastasis to the mouse lungs in vivo involving increased nuclear localisation of β-catenin and suppression of endothelial tight-junction proteins. Phosphorylation of β-catenin in many cell types such as cancer cells results in its proteolytic degradation.^32^ Although a portion of the phosphorylated β-catenin undergoes proteolytic degradation in Akt1-deficient endothelial cells, these cells still have enough of phosphorylated β-catenin to translocate to the nucleus and function as a transcription repressor for claudins. Such a role for the phosphorylated β-catenin has been demonstrated in the normal as well as the tumour endothelial cells.^23, 33^ ShAkt1 HLECs promoted interaction and invasion of PC3 and DU145 prostate cancer cells through their monolayers compared to shControl HLECs in vitro, associated with increased nuclear translocation of β-catenin. Interestingly, reduced expression of claudin-5, ZO-1 and ZO-2 in the shAkt1 HLECs was rescued upon treatment with β-catenin inhibitors ICG001 and IWR-1, which resulted in the attenuation of DU145 prostate cancer cell invasion through shAkt1, compared to a shControl HLEC monolayer in vitro. Although the mRNA levels of claudin-5 in HLECs were significantly lower in shAkt1 HLECs compared to shControl, which is reversed by treatment with β-catenin inhibitors, similar effects of Akt activity and β-catenin inhibition was not observed in the mRNA levels of ZO-1/2 suggesting that unlike claudin-5, ZO1/2 expression as result of Akt1 suppression or β-catenin inhibition is regulated via proteolytic degradation rather than transcriptional modulation. This also confirms our previous observation that Akt1-mediated endothelial-barrier modulation is predominantly through expression modulation of claudin-5 and not ZO-1/2.^14^ Most importantly, in a VECad-Cre-Akt1 mouse model, murine RM1 metastatic prostate cancer cells showed a significant increase in the rate of metastasis to the lungs, but not on the growth of tumour xenografts. Increased prostate cancer metastasis in these mice was significantly inhibited upon treatment with β-catenin inhibitor ICG001. Altogether, these results demonstrate the endothelial cell-specific role of Akt1 in prostate cancer metastasis.

Since Akt phosphorylation was upregulated by VEGF,^34^ a pro-angiogenic molecule that was initially identified as a vascular permeability factor,^35^ Akt activation was widely believed to promote vascular permeability and hence tumour growth and metastasis. In contrast to this dogma, our study using the systemic Akt1 knockout mice revealed increased angiogenesis in response to adeno-VEGF expression in the skin and melanoma tumour implantation.^13^ A major contributing factor to this phenotype was increased vascular permeability in Akt1 knockout mice because of reduced thrombospondin-1 and increased angiopoietin-2 expression, which provided a novel role of Akt1 in endothelial-barrier protection. These results were later confirmed in a different model of VE-cadherin overexpression and clustering in mice where the integral role of tight-junction proteins contributing to vascular protection by Akt1 was identified.^23^ Since then, a number of other researchers also reported the function of Akt1 as vascular protective in various disease models,^36, 37^ demonstrating the integral role of Akt1 on endothelial-barrier and vascular protection in vivo.

An important gap in our knowledge on Akt’s role in vascular permeability vs vascular normalisation in tumours was the mechanisms leading to its differential regulation by various vascular permeability modulators. For example, vascular permeability inducers such as VEGF and TNFα^13, 14, 21, 38^ as well as vascular permeability blockers such as angiopoietin-1 and Robo-4^39, 40^ activated Akt1. Our most recent studies using the VE-Cad-CreAkt1 mice revealed that irrespective of the stimuli, Akt activation has a net endothelial-barrier protective effect^14^ and that the transient vascular permeability effect of VEGF is due to increased Src activity, independent of Akt1.^21^ These studies further informed us the important function of Akt1 to inhibit FoxO and β-catenin transcriptional activities by preventing its nuclear entry, thus protecting the tight junctions via preserving the expression of 20-member claudin family of proteins.^14^ Such an effect of Akt1 on tight-junction protein, claudin-5, in particular, was also previously reported upon VE-cadherin overexpression in endothelial cells.^23^ The latter study also reported cooperation between FoxO and β-catenin in mediating the transcriptional repression of claudin-5. This enabled restoring claudin-5 expression in endothelial cells by treating with either one of the FoxO or β-catenin inhibitors.^14^ In the current study, inhibition of β-catenin resulted in the reversal of claudin-5, ZO-1 and ZO-2 in endothelial tight junctions, and blunted the enhanced ability of PC3 and DU145 cells to migrate through the shAkt1 compared to shControl HLECs indicating the involvement of β-catenin in the process.

Another important candidate downstream of Akt1 activation resulting in β-catenin nuclear translocation is the glycogen synthase kinase-3 (GSK-3).^18, 19^ GSK-3 is known to phosphorylate β-catenin,^18^ thereby dislodging it from the adherens junction and translocating it to the nucleus.^19^ Apart from this, cytosolic β-catenin levels are also determined by the Wnt signalling pathway.^41^ The ability of both transcriptional inhibitor (ICG001) and Wnt signalling inhibitor (IWR-1) to suppress human DU145 cell invasion in vitro and murine RM1 prostate cancer cell metastasis in vivo through the Akt1-deficient endothelium via reduced nuclear translocation of β-catenin and restoration of tight-junction protein expression revealed the importance of total nuclear pool of endothelial β-catenin on prostate cancer metastasis. In prostate cancer cells, the effect of GSK-3 on β-catenin phosphorylation is also dependent on Src-mediated phosphorylation of its tyrosine 218 residues.^18, 19^ On the other end, reduced Akt1 activity in endothelial cells is associated with increased Src activity.^21^ In our study, shAkt1 HLECs exhibited increased GSK-3 tyrosine 218 phosphorylation, increased β-catenin phosphorylation, and its nuclear translocation compared to shControl cells indicating the role of GSK-3 in the process.

Although the effect of β-catenin inhibition on suppressing lung metastasis in VE-Cad-CreAkt1 mice would appear to be a promising strategy to prevent prostate cancer metastasis, our experience with regard to the role of Akt1 in advanced prostate cancer informs us to be more cautious with such an approach. In a very recent study investigating the stage-specific role of Akt1 in early and advanced prostate cancer, we identified that although Akt1 is essential for the oncogenic transformation and tumour growth; its suppression promotes metastasis.^8^ Such a novel role of Akt1 has also recently been shown in breast, liver and non-small cell lung cancers.^42–45^ Unlike endothelial cells, Akt1 deletion in prostate cancer cells although activated by GSK-3, the resultant increase in β-catenin phosphorylation led to its proteolytic degradation.^8^ Hence, we believe that, although inhibition of β-catenin resulted in the prevention of prostate cancer metastasis to the lungs in VE-Cad-CreAkt1 mice, long-term treatment with β-catenin inhibitors may promote epithelial-to-mesenchymal transition specifically in cancer cells and hence metastasis. Nevertheless, this study demonstrates for the first time that Akt1 suppression in endothelial cells will promote prostate cancer metastasis involving β-catenin-mediated suppression of tight-junction proteins such as claudin-5, ZO-1 and ZO-2.

## Electronic supplementary material

Supplemental Figure

## Availability of data and material

This study has no gene arrays or high-throughput screening. All the data related to the study are included within the article and the supplemental material.
