# Supplementary material for: Endothelial Akt1 loss promotes prostate cancer metastasis via β-catenin-regulated tight-junction protein turnover
Source: Br J Cancer. 2018 May 14;118(11):1464–75. doi: 10.1038/s41416-018-0110-1 (PMC5988746; doi:10.1038/s41416-018-0110-1)
Supplement: Supplementary file 1 — Supplemental Figure [file 41416_2018_110_MOESM1_ESM.pdf]

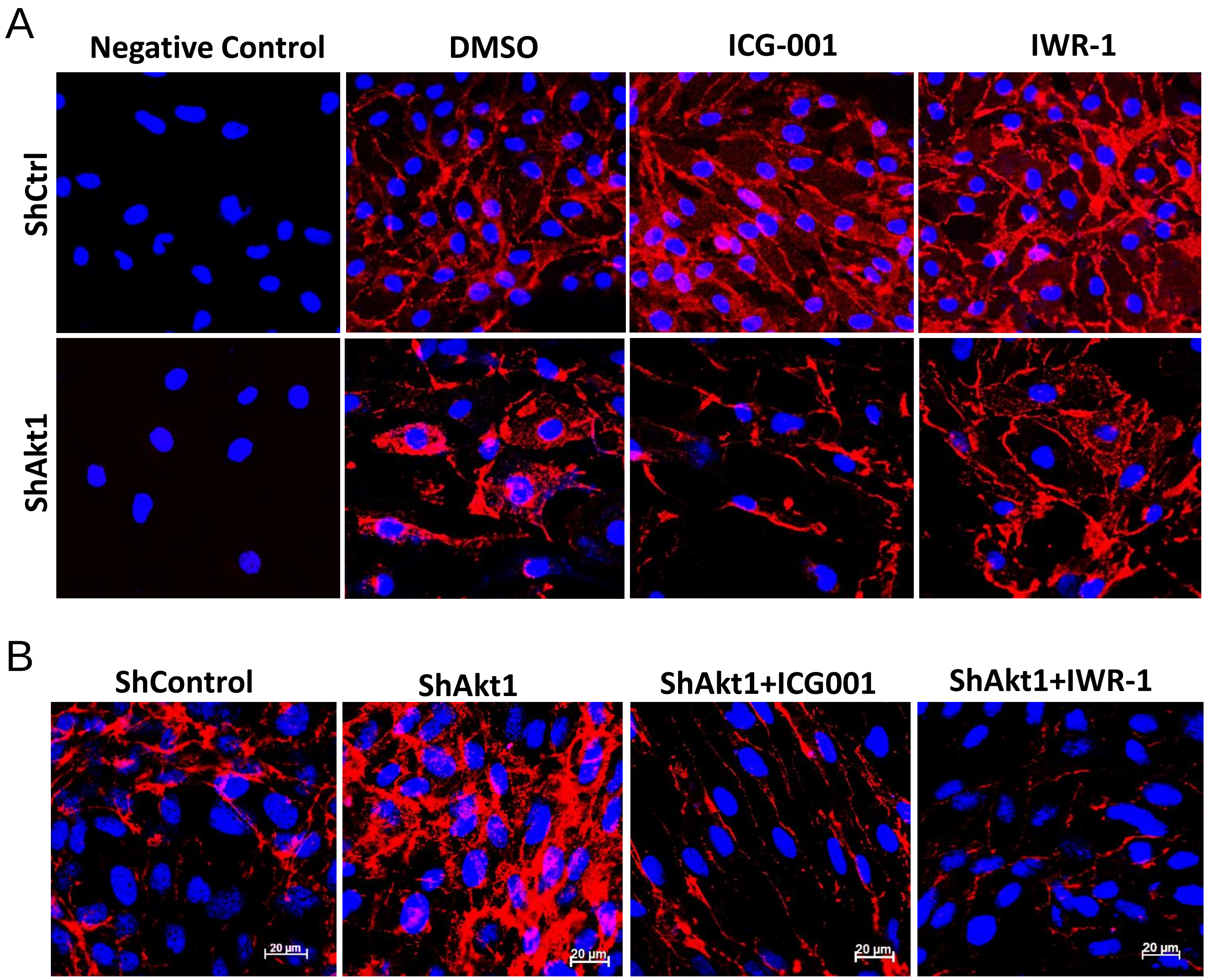

Supplemental Figure 1: Akt1 gene silencing in HLEC increases β-catenin nuclear localization. (A) Representative confocal images of β-catenin and DAPI in ShAkt1 HLEC monolayers treated with control DMSO and β-catenin inhibitors (ICG001 and IWR-1) compared to the negative control where non-immune serum was used as a control (n=6). Red indicates total β-catenin and green indicates DAPI staining. Red indicates total β-catenin and Blue indicates DAPI staining. **(B)** Representative confocal images of pβ-catenin and DAPI stained ShAkt1 HLEC monolayers treated with control DMSO and β-catenin inhibitors (ICG001 and IWR-1) (n=6). Red indicates phosphorylated β-catenin and Blue indicates DAPI staining.
